# Research utilization in municipality nursing practice in rural districts in Norway: a cross-sectional quantitative questionnaire study

**DOI:** 10.1186/s12912-020-00475-1

**Published:** 2020-09-14

**Authors:** Aud Moe, Ingela Enmarker

**Affiliations:** 1 Faculty of Health Science, Nord University, Postbox 1490, N-8049 Bodø, Norway; 2 Centre of Care Research, Levanger, Mid Norway Norway; 3 Faculty of Health and Occupational Studies, University of Gävle, Gävle, Sweden

**Keywords:** Cross-sectional quantitative study, Nursing practice, Research utilization, Rural

## Abstract

**Background:**

Scientific knowledge and theory constitute part of the nurse’s competence and evidence-based nursing practice. To obtain and maintain these skills, nurses require access to research utilization. The aim of the present study was therefore to describe and compare nurses in nursing homes and home-based nursing care and their use of research knowledge in their practice in elderly care in Norwegian rural districts.

**Methods:**

The Research Utilization Questionnaire (RUQ) was employed in cross-sectional quantitative design. One hundred nurses were recruited from ten rural municipalities that participated in the study. Inclusion criteria for participating were registered nurses and employees working in the municipal elderly care service for 6 months or more.

**Results:**

Most participants were younger than 55 years old, worked in permanent jobs, and were educated more than 5 years ago. The result showed that nurses in nursing homes were significantly more positive compared to nurses in home-based nursing care when analyzing all three domains in the RUQ together, as well as for attitudes towards research when testing each domain separated. Overall, each item in the domains revealed opinions that were more positive for nurses in nursing homes. The regression analysis showed that attitudes towards research, as well as availability and support of research utilization predicted the use of research in daily practice.

**Conclusions:**

Positive attitudes, availability, and support for research utilization can contribute to greater use of research in nursing practice and improve the quality of service. Younger nurses’ knowledge about using research should be shared with senior colleagues, who possess much experience in practice. In collaboration, they can develop evidence-based practice by the implementation of research seen in the context of nurses’ experiences, user involvement, and person-centred practice. The i-PARIHS (Promoting Action on Research Implementation in Health Services) framework can be a useful tool in this implementation process.

## Background

There is a trend in several European countries towards increased responsibility for treatment and care shifting from hospitals to municipalities’ health care services [1]. This has led to augmented competence requirements for nurses in municipality health care services [2]. In rural districts, nurses make up a lesser proportion of the health workforce than in urban settings, which makes their role in health care services provision even more significant [3, 4]. Therefore scientific knowledge and theory constitute an important part of the nurse’s competence [5] and evidence-based nursing practice [6].

Research utilization is an area studying the use of research practice [7–9]. Estabrook (p.19) [10, 11] defines research utilization as “the use of research findings in all aspects of one’s work as a registered nurse”. The use of research knowledge is important in raising the quality of care [12, 13], promoting critical thinking [9, 14–16], and reflecting nursing practice [14]. Yet, changes in clinical practice address challenges in transferring research to practice [17].

Access and support are important factors for applying research in practice depending on organizing the service [18], work culture [11, 13, 18, 19] and time [11, 13, 20, 21]. Other factors are knowledge about research utilization [11, 22–25], evidence-based nursing as an intention, and nurses’ confidence in their own ability to apply research [18, 26].

Furthermore, Champion and Leach [27] identified attitudes towards evidence-based practice (EBP) as important in benefitting from research. Research shows various findings about nurses’ attitudes, as studies have found that many nurses have negative attitudes towards the use of research in their practice [23], while other studies found the opposite result [11, 21]. Even if they are positive, some nurses find it difficult to implement research findings in their nursing practice [11]. However, carers in health care service with bachelor’s degrees or higher education have proven to be more positive regarding EBP compared to health care service workers with lower education [28, 29].

In Scandinavia, newly educated Norwegian nurses reported positive attitudes towards research, but few reported using research in their own practice [9]. Swedish nurses reported the same result, i.e. they showed relatively little use of research findings in daily practice in municipality health care services despite their positive opinions [7]. In Norway, many rural municipalities constitute the setting for the use of evidence-based practice in elderly care. Nurses often work alone, both in nursing homes and in home-based nursing care. They are dependent on their own decisions and behaviour [30], especially in home-based nursing care, working at a geographical distance from the administration centre [31].

The setting in which practice takes place seems to be of importance [19, 32]. Nurses in rural municipalities work in different settings [24], such as nursing homes or in patients’ residences. In municipality health care services, Boström et al. [7] showed that greater use of research is provided for those who have access than those with inadequate support and accessibility to research knowledge.

To facilitate the implementation of research utilization the PARIHS group published the PARIHS (Promoting Action on Research Implementation in Health Services) framework, further developed in the i-PARIHS framework for application of the implementation goals through facilitating of an innovation with the recipients in context [33].

Several authors [13, 17, 23] have identified the challenges of using research in practice. However, to the best of our knowledge, only a few of them have addressed their aims to nurses in rural municipality health care services. There is a research gap in exploring differences between nursing practices in home-based nursing care and nursing homes. Knowledge about this can be important to meet the challenges for older patients with comorbidity, considering new and updated knowledge in need of more advanced nursing practices in municipalities whether it is given in patients’ homes or in nursing homes.

Thus, little is known about whether nurses in practice are using updated research findings to provide older patients with safety, quality of care and expertise competence. Therefore the research issues in the present study were: Are there differences in attitudes towards research, in availability and support to implement research findings, and are there differences in the use of research in daily practice between nurses in nursing homes and home-based nursing care? The aim of the present study was therefore to describe and compare nurses in nursing homes and home-based nursing care and their use of research knowledge in their practice in elderly care in Norwegian rural districts.

## Methods

The questionnaire study used a cross-sectional quantitative design [34].

### Participants and procedures

The study population was recruited from ten municipalities representing rural areas: < 5000 inhabitants in central Norway. The inclusion criteria for participating were: registered nurses and employees in the municipality elderly care service for 6 months or more. In these ten municipalities, 100 nurses agreed to participate.

After receiving permission from managers in the municipalities, study information, and the invitation for participation and questionnaire were forwarded to the relevant head administrators of elderly health care departments. In turn, they contacted and informed the nurses about the study. Written information was given together with the questionnaire. The answered forms were returned to the administrator and thereafter returned to the researchers in sealed envelopes. There were two reminders. External drop-outs are unknown because the researchers did not communicate directly with the nurses who met the inclusion criteria.

### Questionnaire

In order to measure the use of research by nurses in practice, the Research Utilization Questionnaire (RUQ) was used. The RUQ was developed by Champion and Leach [27] and further developed by Pettengill, Gillies and Clark and Humphris [35]. The questionnaire consists of three domains: attitudes towards research (12 items), availability and support for the implementation of research findings (8 items), and research use in daily practice (9 items). All items are ranged from one to five on a Likert scale. The questionnaire used in this study was first translated into Swedish [36] and then the items constituting the three indexes were further translated into Norwegian [9]. In Sweden and Norway the questionnaire has been used, e.g., in studies of newly qualified nurses [7, 9, 37]. It has also been used in evidence-based care for the elderly in Sweden [7]. Cronbach’s alpha values for the three research indexes were: attitudes towards research 0.88, availability and support for the implementation of research findings 0.75, and research use in daily practice 0.84, respectively [36]. In this study, the Cronbach’s alpha values were 0.89, 0.72 and 0.76, respectively, for the three domains. In addition, demographics, such as gender, age, seniority as nurse, place of working (nursing home or home-based nursing care) and employment conditions (permanent or temporary employment) were also requested, see Table 1.

**Table 1:** Characteristics of participants working in nursing homes and home-based nursing care in numbers and percent

Demographic data	Nursing homes ***N*** = 47 (%)	Home-based nursing care ***N*** = 53 (%)	Total work setting ***N*** = 100 (%)
*Age*			
20–39 years	23 (47.9)	25 (52.1)	48 (48.0)
40–55 years	22 (47.8)	24 (52.2)	46 (46.0)
56–70 years	2 (33.3)	4 (66.7)	6 (06.0)
*Gender*			
Women	46 (47.9)	50 (52.1)	96 (96.0)
Men	1 (25.0)	3 (75.0)	4 (04.0)
*Seniority as nurse*			
0–5 years	8 (36.4)	14 (63.6)	22 (22.0)
6–10 years	21 (55.3)	17 (44.7)	38 (38.0)
Above 10 years	18 (45.0)	22 (55.0)	40 (40.0)
*Employment condition*			
Permanent	44 (45.8)	52 (54.2)	96 (96.0)
Temporary	3 (75.0)	1 (25.0)	4 (04.0)

### Data analysis

To describe the data, frequencies and percentages were conducted. Differences were tested by Chi^2^ and ANCOVA, and predictions with multiple linear regression analysis. However, before carrying out the statistical analyses, negative items in the RUQ were reversed. Following Wangesteen et al. [9], for the calculations of the three indexes of the RUQ, we added each participant’s score for each domain and then divided the sum within the number of items in each domain respectively. In accordance with Boström et al. [38] and Wangesteen et al. [9], the index of “research use in daily practice” was then dichotomized into research user group score > 3.6 and non-researcher user group. To compare newly graduated nurses (0–5 years) with nurses having more years in the profession (> 5 years), “seniority as nurse” was dichotomized into two groups. In the multiple linear regression analysis, we followed Green’s rule of power, *N* > 50 [39]. The computer program SPSS for Windows version 23.0 was used, and the *p*-value 0.05 was set up for all analyses.

### Ethics

The study was conducted during 2016, and carried out in accordance with the Declaration of Helsinki (World Medical Association, 2008). The nurses were asked to participate by their managers, and were given written information about the purpose of the study that participation was voluntary, and that data would be presented at group level. Completed questionnaire served as the participants’ written consent, and were returned by the managers to the researchers. Thereby, the participants were anonymous by the researchers, and confidentiality was taken care of. Norwegian Social Science Data Services deemed that the need for formal ethics approval was not required. Their response was that because the data have no traceable personal information and the participants were anonymous for the researcher in this project formal ethics approval was not required. We received the project-number 43058.

## Results

The characteristics of the participants working in nursing homes and in home-based nursing care are given in Table 1.

Approximately 95% of the responding nurses working in nursing homes and in home-based nursing care were women, younger than 56 years old, and working in permanent jobs. The majority of the nurses were educated 10 years ago or earlier.

The first step in the analysis was to compare the use of research among nurses in nursing homes and in home-based nursing for the three domains together. As previous research indicated that newly educated nurses (0–5 years) could be more positive towards research utilization than those earlier educated, seniority as nurse was employed as a covariate in the analysis. The statistical analysis showed a significant difference in the RUQ, (F (1, 97) = 4.14, *p* = .045). See Table 2.

**Table 2:** ANCOVA testing of Total mean of RUQ between Nurses in nursing homes and Nurses in home-based nursing care with Seniority as nurse as covariate

Source	df	SS	MS	F	*p*
Working setting	1	1.145	1.145	4.143	.045
Seniority as nurse	1	483	483	1.749	.189
Error	97	26.817	276		
Total	100	1190.759			

*Note.* Seniority as nurse (< 5 years and > 5 years); *SS* Sums of square, *MS* Mean of square

That is, nurses who worked in nursing homes were more positive towards research utilization than were nurses in home-based nursing care. Further, when the analysis was divided into the three domains each, there was a significant difference in “attitudes towards research”, (F (1, 97) = 3,97, *p* = .049). See Table 3. The results showed the same pattern nurses in nursing homes were more positive than in nurses in home-based nursing care. Means and standard deviations for each domain are shown in Table 4.

**Table 3:** ANCOVA testing of the domain of Attitudes towards research between Nurses in nursing homes and Nurses in home-based nursing care with Seniority as nurse as covariate

Source	df	SS	MS	F	*p*
Working setting	1	1.688	1.688	3.966	.049
Seniority as nurse	1	220	220	517	.474
Error	97	41.289	.426		
Total	100	1579.722			

*Note.* Seniority as nurse (< 5 years and > 5 years); *SS* Sums of square, *MS* Mean of square

**Table 4:** Frequencies of agreements (strongly agree/agree) in RUQ among nursing homes and nurses in home-based nursing care with Chi^2^ analysis and mean and standard deviation

Index M (SD)	Item	Nursing homes n (%) (*N* = 47)	Home-based nursing n (%) (*N* = 57)
A. Attitudes towards research *Nursing homes* 4.05 (0.64) *Home-based nursing* 3.80 (0.66)	*A1. I wish to change my practice to make it based on research*	*34 (72.3)*	*34 (64.2)*
	*A2. I want to base practice on research*	*32 (68.1)*	*30 (56.6)*
	*A3. Clinical practice should be based on research*	*42 (89.4)*	*44 (83.0)*
	*A4. Participating in research is waste of time*	*3 (6.4)*	*5 (9.4)*
	*A5. Understanding research helps professionally*	*42 (89.4)*	*41 (77.4)*
	*A6. I think research is interesting*	*44 (93.6)*	41 (77.4)*
	*A7. Research is stimulating*	*43 (91.5)*	*42 (79.2)*
	*A8. Research is understandable*	*40 (85.1)*	*38 (71.7)*
	*A9. Research is dull, boring project*	*6 (12.8)*	*11 (20.8)*
	*A10. It is not relevant to use research findings in day-to-day practice*	*6 (12.8)*	*11 (20.8)*
	*A11. Basing clinical practice on research is time-saving*	*23 (48.9)*	15 (28.3)*
	*A12. Research findings are too complex to use in practice*	*16 (34.0)*	*23 (43.0)*
B. Availability and support to implement research findings *Nursing homes* 3.27 (0.62) *Home-based nursing* 3.13 (0.60) C. Research use in daily practice *Nursing homes* 3.22 (.68) *Home-based nursing* 3.01 (.65*)* Total RUQ index	B1 *The clinical team I work with supports research utilization*	*21 (44.7)*	*24 (45.3)*
	B2 *My unit manager supports research utilization*	*26 (55.3)*	*28 (52.8*
	B3 *The quality of research is not so good that it can be used in practice*	*21 (44.7)*	*32 (60.4)*
	B4 *I have access to research findings where I work*	*28 (59.6)*	*27 (50.9)*
	B5 *I have time to read to read about research while I am on duty*	*12 (25.5)*	*10 (18.9)*
	B6 *Research is performed in my work*	*17 (36.2)*	08 (15.1)*
	B7 *Research is performed in the community*	*17 (36.2)*	*21 (39.6)*
	B8 *Education in research is carried out in the community*	*25 (53.2)*	*21 (39.6)*
	C1 *I base my practice on research*	*31 (66.0)*	*27 (50.9)*
	C2 *My clinical practice is based on research*	*31 (66.0)*	*29 (54.7)*
	C3 *I do not use research in day-to-day practice*	*17 (36.2)*	*25 (47.2)*
	C4 *I use research findings in my clinical practice*	*30 (63.8)*	*27 (50.9)*
	C5 *I apply research findings in my clinical practice*	*21 (44.7)*	*20 (37.7)*
	C6 *I help others to apply research in clinical practice*	*24 (51.1)*	*18 (34.0)*
	C7 *I use research to guide my clinical practice*	*21 (44.7)*	*17 (32.1*
	C8 *I cannot apply research findings in my clinical practice*	*8 (17.0)*	20 (37.7)*
	C9 *I seek out research related to my clinical practice*	*26 (55.3)* 3.51 (.52)	*19 (35.8)* 3.31 (.53)

Note. * = *p* < .05

Moreover, in Table 4, the frequencies and percentages of agreed responses (strongly agree/agree) for all items of the RUQ are given. When identifying the frequencies of agreements for the domain “attitudes towards research”, a majority of the nurses in both settings agreed that the *clinical practice should be based on research.* The Chi^2^ analyses revealed significant differences between nurses in nursing homes and nurses in home-based nursing care in their views that basing clinical practice on research is time-saving, *X*^*2*^(1, *N* = 100) = 4.502, *p* = .034 as well as whether research is interesting, *X*^*2*^ (1, *N* = 100) = 5.164, *p* = .023. That is, in those items, the nurses working in nursing homes had a more positive attitude compared to the nurses in home-based nursing care.

In “availability and support to implement research findings”, above half (50%) of the nurses considered they had access to research findings at work, but the percentage of time to read about research on duty was lower, about 25%, respectively 19%. When analysing their ratings regarding whether research was performed at their work, there was a significant difference in the proportion of agreements, 17 nurses in nursing homes agreed, and eight nurses in home-based nursing care, *X*^*2*^(1, *N* = 100) = 5.901, *p* = .015).

In the domain “research use in daily practice”, the nurses in both work settings deemed they used research findings in their clinical practice (30%, respectively 27%). A significant difference emerged in the item “I cannot apply research findings in my clinical practice”. That is, fewer nurses in nursing homes agreed with that statement than did nurses in home-based nursing care, *X*^*2*^ (1, *N* = 100) = 5.302, *p* = .021.

Overall, the Chi^2^ analyses indicated that nurses in nursing homes showed more positive attitudes, they had better availability and support to implement research findings, and more often, they used research in daily practice than did the responding nurses in home-based nursing care.

Finally, to examine if “attitudes towards research” and “availability and support to implement research findings”, as well as, work setting and seniority as a nurse could predict the research use in daily practice, a multiple linear regression analysis was conducted. The model indicated a significant effect, (F (4,95) = 17.36, *p* < .000, *R*^2^ = .42). The analysis revealed that attitudes towards research significantly predicted the use of research, as well as availability and support to implement research findings. Neither working in nursing homes or in home-based nursing care, nor seniority as nurse (< 5 years; > 5 years) could predict research utilization (See Table 5).

**Table 5:** Predictors for Research use (RUQ index)

*Independent variable*	*B*	*SE B*	β	*t*	*p*	*95% CI for B*
Attitude index	.422	.091	.415	4.621	.000	.241–.603
Availability index	.326	.097	.298	3.364	.001	.134–.518
Working setting	−.082	.107	−.061	−.765	ns	−.294–.131
Seniority as nurse	−.217	.127	−.135	−1.714	ns	−.469–.034

## Discussion

The aim of the present study was to describe and compare nurses in nursing homes and home-based nursing care and their use of research knowledge in their practice of elderly care in Norwegian rural districts by using the RUQ questionnaire. The result showed significant differences between nurses in nursing homes and home-based nursing care in the RUQ when including all three domains, as well as, for attitudes towards research when analysing each domain separately. Chi^2^ analyses for each item in the domains revealed that nurses in nursing homes reported opinions that were more positive than did nurses in home-based nursing care. Attitudes towards research, as well as availability and support of research utilization, did predict the use of research in daily practice.

The significant differences between nurses working in nursing homes and those working in home-based nursing care could influence the implementation of research differently. In the implementation process, the i-PARIHS framework can be used for successful implementation and consist of components innovation, recipient, context and facilitation. Facilitation represents the active element to assess, align and integrate the other three constructs [33].

### The innovation construct

New and updated knowledge is important for patient safety and quality of care for older persons with comorbidity. Overall, nurses in both nursing homes and in home-based nursing care indicated positive attitudes towards research and agreed that the clinical practice should be based on research. However in some issues, our findings showed significant differences, i.e. nurses in nursing homes had attitudes that were more positive towards using research in their work than the responding nurses in home-based nursing care. The results in previous studies are not coherent about nurses’ attitudes towards using research. Some studies do not confirm that nurses have this positive attitude towards research findings [23], while others confirmed the result in the present study [11, 21].

The differences in the use of research in nursing homes and home-based nursing care can depend, in that some nurses seem to be satisfied with being passive in their role. They are leaving evidence in care to other professionals, e.g. physicians. Although the use of research is limited, overall, nurses have a desired intention to include evidence in their work to update their nursing through the latest research [26]. However, Estabrooks et al. [40] found that nurses do not commonly use the term “research utilization”, but gave many examples of using research when moving from indirect to direct care of participants. Other studies found there was a lack of knowledge about how to increase research utilization [11, 23, 24]. People incorporate evidence in different ways, which involves adapting ([33], p. 4).

When comparing nurses in home-based nursing care and nursing homes, we found that only a few nurses in both work settings felt that research is dull and boring and not relevant to use in daily practice. According to other studies, nurses may have positive attitudes towards research, but still do not use it in their practice [21, 24]. In this study, many nurses agreed on the importance of using research in their own practice, but not in their daily work. Organizational changes in municipalities and health service have led to greater challenges in caring than before, as nurses’ earlier experiences gained greater recognition. This requires a change in the view of different forms of knowledge and the need to renew the knowledge that in today’s environment shows inadequate competence among nurses in municipal health care [2]. This include that evidence is generated from practice [33]. In developing clinical practice, nurses are important influencers.

### The recipient construct

In the present study, the recipients were nurses working in nursing homes and in home-based nursing care. Most of them were younger than 56 years old, worked in permanent jobs, and the majority were educated more than 5 years ago. According to Harvey and Kitson [33], the recipients affect and influence the implementation on both individual and collective team level in supporting or resisting the innovation. Among the nurses in home-based nursing care, 43% thought research findings were too complex to use in practice, while 34% of the nurses in nursing homes agreed. This may be explained by poor knowledge of evidence-based practice and by poor English comprehension [23]. Developing skills in the finding, reading and understanding of research can make research less complex [24].

There was no significant difference in attitudes among senior and junior nurses in this study. Most nurses were educated more than 5 years ago. This means that most of them did not have EBP as a part of their education and needed education and training in finding and reading research, which is a prerequisite for using and implementing research in nursing practice. The study of Gardner et al. [24] showed that senior nurses had a more positive orientation to research. Conversely, Gustafsson et al. [26] found that older nurses expressed that EBP was easier to practice for younger nurses who recently had used it in their training. Newly educated nurses can be seen as a resource on a micro level in the organization with knowledge and skills in using research in their work.

The relationship between colleagues is important to foster collaboration on implementation. Interpersonal relations and environmental factors are reported to influence physical and psychological dimensions [20]. When working alone, as in home-based nursing care, there can be a lack of collegial interpersonal relations among employees. Accordingly, Lea [4] considered that there could be a challenge with few professional nurses, which may be due to recruitment problems [41], but also limited communication between the employees in rural areas is a challenge [4]. Limited collegial collaboration can affect the motivation to implement knowledge in practice, as recipients, in this case nurses, construct and encompasses the people who are affected by and influence implementation at both individual and collective team level and consider the impact individuals and teams have in supporting innovation [33].

The application of knowledge in nurses’ practice can also be influenced. Regardless of the arena, collaboration is needed at the meso level, in the local development of the service, and at the macro level through planning and facilitation for collaboration with user organizations in developing a good patient-safety service. At the meso- and macro level, contextual factors are recognised as important considerations in innovation; they incorporate evidence in different ways, involving adapting the original evidence in some way to suit their particular situation [33]. The nursing practice has been referred to as the process of shared decision-making between the practitioner, patient and significant others based on research findings, patient’s expertise and preferences, clinical expertise and other information [42]. Contextual balancing of knowledge explained how nurses dealt with their main concern, how to determine what types of knowledge they could trust and how to combine different types of knowledge, such as knowledge from their own experiences, from science and from the patient. This resulted in nurses combining their evidence-based practice with a sense of control in the actual situation [8].

### The context construct

Research knowledge is published on a large scale, but changes in clinical practice do not have the same progression [21, 25, 26]. Studies show that research is, to a limited extent, used in nurses’ municipal practice [9] and confirm the results in our study. Nurses in rural areas work with a relatively large elderly population. The treatment and care of older patients is complex and knowledge-intensive [30, 31]. In this study, the setting was municipality care represented by home-based nursing care and nursing homes.

Despite positive attitudes towards research, many nurses do not feel competent to find, consider and use research [11, 24, 26]. In addition, there are barriers like poor knowledge, limited motivation and little trust in success [23]. A study by Thompson et al. [20] showed that lack of time is the most reported barrier to research utilization, e.g. lack of time to read research reports while on duty. Stavor et al. [11] found that nurses experienced time was taken away from patients. This may confirm the results from the home care nurses who did not consider it time-saving to use research knowledge. However, nurses in nursing homes agreed to a higher extent that clinical practice based on research is time-saving. This revealed significant differences between nurses in nursing homes and in home-based nursing care regarding the time-saving possibilities of basing clinical practice on research.

In the present study, access and support were reported to be stronger in nursing homes than in home-based nursing care. Nurses in nursing homes are stationary, while nurses in home-based nursing care are mobile and have limited access to office facilities or support from colleagues and leaders [31]. A review study emphasized the importance of leadership style and supportive management to increase the quality of care in nursing homes [43]. Nurses who reported access to research-related resources reported more use of research than did nurses without such resources [38].

The setting differs in nursing homes and home-based nursing care, with a special need for planning, available mobile technology and support for nurses in home nursing to assure the quality of their knowledge. Context may be defined in terms of resources, culture and leadership [33]. In this study, resources and leadership may be at a distance from the workers, which is special in home-based nursing care, and the culture can be individual-oriented with no meeting places for collective collaboration on changes in the organization.

Evidence from Lea and Cruickshank [4], Bennett, Barlow*,* Brown and Jones [44] and Ostini and Bonner [45] indicates that organizational pressures and skill mix are of concern in rural districts. At the same time, more knowledge is required in municipal care [2]. With organizational changes and more responsibility for advanced treatment and care, the focus should be on the complex situation of the older persons with comorbidity [1].

In rural practice, Lea and Cruickshank [4] found that newly graduated nurses were socializing to practice. Their experiences in the first months of rural nursing practice were significantly influenced by the culture of the ward environment, the workload and the level of responsibility. This is found to be a significant association between work culture and quality of care [43].

Other factors that may affect the context in home nursing care are the patients’ home conditions, which are very different between individual patients [31] and at a distance from an institution with necessary equipment available and colleagues working in the same building. Even if nurses’ knowledge and attitudes are present, the practical conditions can make it difficult to practice EBP. This is a challenge for individual nurses to make the best decisions based on research knowledge and nurse experiences, in collaboration with the patient.

### The facilitation construct

To facilitate the innovation process is to activate the other main through assessing and responding to characteristics of the innovation and recipients within their contextual setting [33]. In this study attitudes towards implementing research in practice were largely positive, but the nurses had limited knowledge about finding and using research in practice. Brown, Wickline, Ecoff and Glaser [46] describe nurses’ practices, knowledge and attitudes related to EBP as limited by organizational barriers in the form of nurses’ autonomy, learning opportunities, cultural building and access to resources. Despite this, EBP was a desired intention and mission of the nurses themselves [26].

Evidence-based competence is needed in planning, assessment, health promotion, risk management and clinical activities as part of nurses’ work. That is, it is necessary knowledge of the nurses but also managers who are often facilitators at different levels in the organization. Attitudinal and ethical competence is needed to be able to recognise, reflect and solve ethical dilemmas and to meet the older person with respect [47]. Chronically ill older people in rural municipalities have not only sought theoretical knowledge but also the skills and attitudes of nurses [48]. EBP requires reflection and customization to the context [9, 14, 15, 18], and reflected nursing practice [14]. In addition to an enthusiastic and critical team, high-quality care, shared visions and willingness to learn are important facilitators in EBP [23].

A review study found that competence makes a difference in the quality of care. Long-term care settings with advanced nursing practice had lower rates of depression, urinary incontinence, pressure ulcers, restraint use, and aggressive behaviour. More residents in these settings experienced improvements in meeting personal goals, and family members expressed more satisfaction with medical services [12]. Nurses in nursing homes often work in a “mini-hospital” without doctors or nursing colleagues around [47]. This requires competence to find and apply research in rural nursing practice. According to Benner et al. [42], competence is in three areas: knowledge and science, skills and clinical reasoning, and ethical attitudes and formation.

In a study interviewing head nurses, Johansson et al. [21] found that despite positive attitudes among head nurses towards EBP, they did not have time themselves during the working day to find relevant research but believed they supported the nurses in reading research to improve quality in care. Innovation aims to improve the quality that is influenced by the nurses’ expertise and facilitation through flexible management and feedback through the process to tailor their approach to the particular issue, setting and people involved ([33], p. 6).

In recent years, there have been comprehensive organizational changes in the municipalities, and at the same time, health services also are constantly changing to meet the authorities’ requirement to provide knowledge-based practice [49]. Organizational changes at the national, regional and municipal levels make it difficult for municipalities to meet all requirements and expectations from governments and the population. This requires a role, the facilitator, and a set of strategies and actions to enable implementation [33] so older persons can receive advanced nursing both in their homes and in nursing homes.

Our results showed that positive attitudes towards research, availability and support could predict the use of research. Research utilization is a part of EBP and necessary to provide nursing practice of high quality. Many nurses need training to find and read research results to be stimulated, understand research and work professionally [11, 25, 38]. Accordingly, Eizenberg [50] found that education, skills in locating various research sources, as well as support and knowledge based on scientific knowledge predicted evidence-based nursing. DuGan [25] found a positive effect by training in EBP in rural districts. There was a significant difference in increased knowledge of identifying gaps in practice, finding research articles, critical analyses and applying information to individual practice [11]. In an Australian intervention study, Gardner et al. [24] found that nurses in rural areas lack the knowledge to search and understand research. Later, in the intervention process, implementing a training program, nurses working in various settings in rural areas developed a good understanding of journal articles. In Norway, there is a learning network all over the country to focus on and implement EBP [51]. This network gives a possibility to strengthen the EBP in elderly care.

### Strengths and limitations

The strength of this study was the focus on research utilization in rural nursing care. This is expressed as “attitudes towards research”, “availability and support to implement research findings” and “research use in daily practice”. This is a little-explored area, especially in elderly care services in rural areas. The RUQ is validated in a derived measure of research utilization by nurses [52].

The cross-sectional design is appropriate to describe the status of the RUQ at a fixed time. The sample represents nurses in ten small municipalities, both from nursing homes and home-based nursing care, which cover the health service of ill older people in the municipal where a lot of treatment and care are taking place. A limitation could be that the participants were not randomly selected for this study.

Access to the selection was made through managers for the services. On the one hand, a direct contact with participants, without leaders could contribute to a larger selection. On the other hand, participation and reminders from managers could facilitate participation in the project. Alternatively, anonymous completion of the questionnaire can enhance sincerity and participation. As access to the participants occurred via the managers, it was not possible to directly remind participants to reply to the forms.

The sample of nurses in home-based nursing care (*n* = 53) and nursing home (*n* = 47) was small, but the sample number was within the critical limit [53].

## Conclusion

The results showed significant differences between nurses in nursing homes and home-based nursing care in the RUQ when all three domains were included, as well as for attitudes towards research when analysing each domain separately. Positive attitudes, availability, and support for research utilization can contribute to greater use of research in nursing practice and improve the quality of service for older patients in rural districts. Through collegial teamwork, younger nurses’ knowledge about using research should be shared with senior colleagues who possess much experience in practice. In collaboration, they can develop EBP by the implementation of research seen in the context of nurses’ experiences, user involvement, and person-centered practice. The i-PARIHS framework can be a useful tool in this implementation process.

## Data Availability

The datasets used and analyzed during the current study are available from the corresponding author upon reasonable request.

## Abbreviations

EBP: Evidence-based practice
RUQ: Research Utilization Questionnaire
